# Data-Driven Distributed Energy Management in Interconnected Smart Grids/Microgrids: A Critical Review of ADMM and Related Optimization Algorithms

**DOI:** 10.3390/s26123620

**Published:** 2026-06-06

**Authors:** Muhammad Jamshed Abbass, Robert Lis

**Affiliations:** Department of Electrical Engineering, Wroclaw University of Science and Technology, 50-370 Wroclaw, Poland; muhammad.abbass@pwr.edu.pl

**Keywords:** data-driven energy management, distributed optimization, smart grids, interconnected microgrids, ADMM (Alternating Direction Method of Multipliers), Distributed Energy Resources (DERs)

## Abstract

Microgrids are increasingly recognized as transformative and crucial constituents within advanced smart grid systems. This study introduces a decentralized energy management approach for interconnected microgrids that leverage renewable energy sources such as wind and solar, alongside distributed energy generators and storage mechanisms. An energy coalition manager (ECM) plays a key role in facilitating each microgrid’s integration to optimize power exchanges, enhance data communication, and reduce costs. The alternate-direction multiplier method is adapted to address optimization challenges, incorporating modifications to develop a censored version that enhances communication efficacy. This refined approach involves the exchange of information among neighboring entities, evaluated against a preset threshold. Through this precise comparison, ECMs strategically reveal their local variables to ensure convergence towards an optimal solution. A detailed case study was conducted to assess the performance, efficiency, and scalability of both methodologies comprehensively.

## 1. Introduction

A distributed network, which generates and consumes electricity through localized facilities near the demand sites, is progressively supplanting the conventional centralized models of large-scale electricity generation, transmission, and distribution, aiming to mitigate rising concerns about environmental degradation [1]. Nevertheless, the unpredictable nature of renewable energy generation can present operational challenges if not efficiently managed, particularly regarding system power balance. A viable solution to these challenges is the microgrid, a compact distribution system (DS) for electric power that integrates various distributed generation units and loads [2]. These units can be both renewable and controllable, incorporating energy storage systems. Microgrids have emerged as a practical strategy for integrating renewable energy sources and energy storage systems for both economic and reliability purposes [3,4]. They operate safely and efficiently in grid-tied and island modes, allowing autonomous management [5]. In island mode, without assistance from the main grid, a microgrid must maintain an equilibrium between energy supply and demand [6]. A multi-microgrid system (MGS) presents advantages due to the limitations of individual microgrids in terms of energy production and storage capacity. The motivation for this study is to tackle the increasing complexity in managing interconnected microgrids with high penetration of renewable energy sources, where uncertainties, communication constraints, and data privacy issues diminish the effectiveness of existing approaches. Distributed optimization techniques, especially ADMM-based methods, have been widely investigated; however, they usually require heavy communication between agents, which may limit scalability and efficiency in practice. To address these challenges, this paper proposes a data-driven distributed energy management framework with a censored ADMM mechanism for reducing redundant data exchange between neighboring microgrids. The novel aspect of the proposed approach is that it improves communication efficiency without any sacrifice of the convergence performance and reliability of the system. Moreover, the main contribution of this work is the development of a decentralized coordination strategy through an energy coalition manager (ECM), which enables cost-effective operation, enhanced privacy, and scalable integration of distributed energy resources. This interconnection aids in safeguarding against extensive blackouts and enhances control resilience. By sharing spinning reserves or importing cost-effective energy from neighboring microgrids, an MGS facilitates the integration of renewable and storage resources, thereby reducing operational costs [7]. Two primary approaches to MGS energy management exist: centralized and distributed methods. In centralized energy management, a central controller collects data regarding loads, grid conditions, and generation [8]. The controller employs centralized methodologies such as genetic algorithms, the lambda iteration method, and particle swarm optimization to determine optimal solutions [9,10]. Moreover, as referenced in [11,12,13], we explored potential risks associated with the robustness of centralized algorithms in microgrids. In the dynamic landscape of multi-microgrid (MMG) configurations, the authors of [14,15,16] advocate for an optimal concentration of generating resources, a critical factor in shaping the future of energy distribution. These pioneering microgrids are not merely potential contributors; they are emerging as transformative pillars within the advancing smart grid infrastructure. Our research proposes an advanced distributed energy management (DEM) strategy for interconnected microgrids, employing wind and solar energy, augmented by distributed energy storage systems (ESS) and distributed generators (DG).

Microgrids, facilitated by an energy coalition manager (ECM), are integrated to enhance information exchange, optimizing power transactions while minimizing costs. We apply the alternate-direction multiplier method (ADMM) for iterative optimization, introducing a censored variant to boost communication efficiency. This advanced improvement assesses the information shared among neighboring entities against a specified threshold. Following this critical analysis, ECMs disclose their local variables, leading to an optimal solution. An extensive case study was undertaken to thoroughly evaluate the performance, efficiency, and scalability of both methodologies. The centralized model aggregates global data at a single hub, offering a comprehensive view but potentially leading to issues such as privacy breaches, tampering, excessive communication load, and single-point failures. Consequently, distributed optimization, known as DEM, is increasingly regarded as an effective solution to these challenges. DEM is at the forefront of rigorous investigations aimed at enhancing smart grid privacy and productivity [17]. According to reference [18], a distributed and hierarchical architecture can significantly accelerate microgrid energy dispatch (ED) operations, though the proposed solution is limited to a star interconnection topology (SIT). A novel distributed energy trading approach is meticulously detailed in [19], with emphasis on dynamic energy exchange between island MGs. An advanced algorithm, showcased in [20], is founded on fully distributed consensus, designed for efficient energy management in island MGs. Addressing the critical challenges faced by MGs in emergency departments, the authors of [21] propose a groundbreaking consensus-based distributed solution technique. An insightful examination of dispatchable generation and demand response through a distributed consensus framework reveals opportunities for boosting social welfare, as discussed in [22]. Pioneering the application of multi-agent system (MAS) technology, ref. [23] introduces a distributed control algorithm to improve energy resource optimization in island MGs. To reduce the strain on smart grid data exchange infrastructures, ref. [24] adopts an emergency department problem optimization strategy, employing the innovative concept of event-triggered transmission. The introduction of an ideal method for MG control using a fully distributed dispersion strategy is explained in [25]. A contemporary DEM strategy for multi-MG systems is presented in [26], addressing real-time challenges. For complex emergency room issues, the alternate-direction multiplier method (ADMM) is employed as a pivotal distributed control protocol, decomposing optimization problems into manageable components [27,28]. The Alternating Direction Method of Multipliers (ADMM) is recognized for its exceptional applicability to large-scale distributed computing infrastructures and multi-agent networks [29]. This technique eliminates the requirement for a centralized controller during computational processes, allowing local controllers to collaboratively determine the optimal solution via their decentralized optimization framework [30]. Despite its rapid convergence, ADMM entails considerable communication requirements. The core principle of the censorship strategy presented in [31] involves filtering out less significant information according to a predefined threshold, thus reducing communication overhead. In this paper, an integrated architecture for interconnected multi-microgrids (MMGs) is proposed, efficiently embedding renewable energy (RE) sources and energy storage systems (ESS). The proposed framework facilitates the coordinated operation of microgrids (MGs) via active information exchange, mainly focusing on optimal power sharing by means of advanced energy management controllers (ECMs). We adopt an optimization approach using the Alternating Direction Method of Multipliers (ADMM), where a virtual control agent in each ECM allows for decentralized coordination to minimize the overall operational costs. The novelty of this work is to develop a communication-efficient distributed optimization scheme by extending the conventional ADMM to a censored variant, which can greatly reduce data exchange and maintain convergence performance. The main contributions of this work are summarized as follows. (i) Designing a fully distributed ADMM-based energy management framework for optimal resource scheduling in grid-connected MMGs. (ii) Improving the standard ADMM with a censoring mechanism for balancing communication costs and computational efficiency. (iii) Conducting a comprehensive case study revealing the trade-offs between communication overhead and convergence behavior, while achieving as much as a 50% reduction in operational costs associated with distributed generation, storage, and energy procurement.

The manuscript is organized as follows: Part II outlines the energy management framework, Section 2 covers the modeling of Microgrid System Model, Section 3 details the Distributed Censored ADMM Formulation, Section 4 presents the simulation case study, and Section 5 concludes.

## 2. Microgrid System Model

This section is about the architecture of the energy-sharing network. This section also defines the rudiments of the network model.

### 2.1. Architecture of the Energy Sharing Network

A microgrid’s architecture includes a network of distributed generators, loads, and renewable energy (RE) sources.

**Figure 1:**
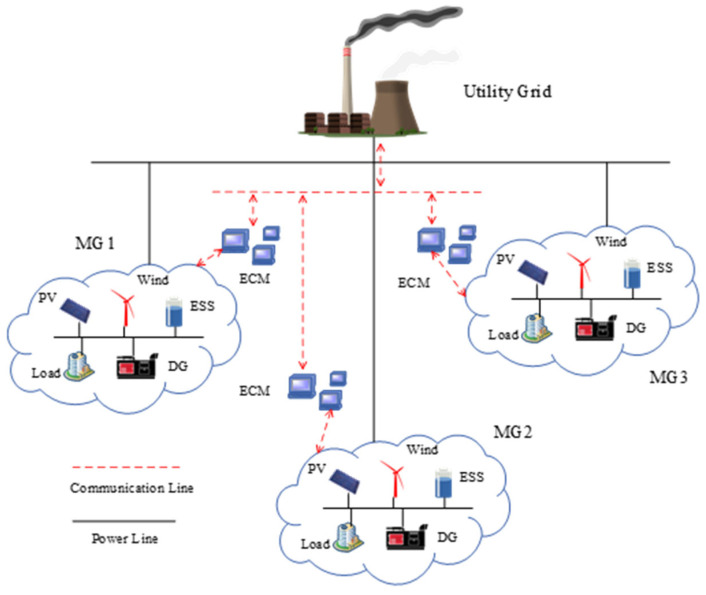
Proposed architecture of an energy-sharing framework in networked microgrids.

As illustrated in Figure 1, each microgrid acts as a distributor within a distribution system, capable of buying or selling electricity to other microgrids (MGs). The bidirectional communication network, indicated by the red dotted lines, can be either wireless sensors or wired infrastructure. Solid lines depict the electric power network. If the energy purchase price for an MG exceeds the selling price, it provides a means to increase the usage of renewable energy by the MG. Additionally, it is assumed that each microgrid has an energy coalition manager (ECM) responsible for data sharing and reducing operational costs. Microgrids are incentivized to minimize line losses by obtaining as much electricity as possible from nearby sources rather than the utility grid. Nonetheless, if there remains a shortfall, energy can be exchanged with the utility company.

(a) Utility Grid

In a multi-MG system, the MGs collaborate to supply their load demands. For instance, if one MG load demand is higher than its generated power, it will collaborate with adjacent neighboring MGs to meet energy demand. In a worst-case scenario, when the multi-MG system is unable to shelter the energy demand, then it will access the utility grid to acquire the obligatory energy demand. On the contrary, if the generated power is higher than the load demand of a multi-MG network, the multi-MG would sell back to the utility grid for generating revenue. Hence, the utility grid’s cost of electricity at time t can be delineated as:

(1) $$C_{G}(t)=\left\{\begin{array}{c} \alpha_{G}x_{G}(t{)}^{2}+\beta_{G}x_{G}(t)+\gamma_{G},x_{G}(t)>0\\ \sigma x_{G}\left(t\right),\;\;\;\;\;\;\;\;\;\;x_{G}(t)<0 \end{array}\right.$$

where $x_{G}(t)$ is the net electric power of the multi-MG system at time t supplied or bought by the utility grid, $\left(\alpha_{G},\beta_{G},\gamma_{G}\right)$ are the generation cost coefficients, and σ is the unit tariff of MG’s renewable energy selling to the utility grid.

(b) Battery Lifetime Models (BESS)

Battery lifetime is reduced after a specific number of charging and discharging cycles. The lifetime loss l of a battery energy storage system can be portrayed as:

(2) $$l=\frac{F_{o}}{F_{C,tot}}\;$$

where l is the calculated lifetime loss percentage of battery ESS, and $F_{o}$ is the effective cumulative throughput in a certain period. The total effective cumulative throughput in a complete lifetime cycle is denoted as $F_{C,tot}$.

Moreover, the battery’s state of charge (SOC) determines the value of the actual capacity $F_{o}^{*}$ and the effective cumulative capacity $F_{o}$. The relationship between both variables can be defined as:

(3) $$F_{o}=\tau_{SOC}F_{o}^{*}$$

In Equation (3), $\tau_{SOC}$ is the effective weight, and it can be defined as [32]:

(4) $$\tau_{SOC}=\alpha_{ESS}*SOC+\beta_{ESS}$$

where $\alpha_{ESS}$ and $\beta_{ESS}$ are the empirical parameters. $F_{o}$ is depicted when *SOC* changes from $SOC^{\min}$ to $SOC^{max}$ during the operation of discharging/charging. It can be expressed as:

(5) $$SOC^{\min}\leq SOC\leq SOC^{\max}$$

where $SOC^{\min}$ and $SOC^{\max}$ are the minimum and maximum limits of SOC. The limits of the power output of ESS are defined as:

(6) $$x_{ESS}^{chr,\max}\leq x_{ESS}\leq x_{ESS}^{dis,\max}$$

where $x_{ESS}^{chr,\max}$ and $x_{ESS}^{dis,\max}$ are the maximum limits of allowed charging and discharging power. The $x_{ESS}$ is positive for discharging and negative for charging. The SOC value of a discrete time (k=t+Δt) can be represented as:

(7) $$SOC_{k}=SOC_{t}-x_{ESS}*\frac{\Delta t}{U}$$

where *U* is the battery capacity, note that the charging and discharging efficiency is assumed to be 100% here to simplify the lifetime model.

By substituting Equations (3), (4), and (7) in (2), the lifetime cost as a function of $x_{ESS}$ can be expressed as:

(8) $$C_{ESS}(x_{ESS})=\eta \;.\;l=\eta E_{o}^{*}\frac{\alpha_{ESS}(USOC_{t}-x_{ESS}\Delta t)+U\beta_{ESS}}{U\;E_{C,tot}}$$

where η is the investment cost of battery ESS.

In this study, the cost models of battery energy storage systems (BESS) and renewable energy sources are explicitly considered in the objective function. The cost of BESS is modeled based on lifetime degradation and investment cost, with charging and discharging cycles contributing to operational expenses. Renewable generation, such as photovoltaic and wind generation, is assumed to be negligible in fuel costs, but their variability influences system scheduling and may indirectly influence operational costs due to reliance on backup generation or storage. It is very beneficial to the system to integrate BESS and renewable sources. In particular, BESS increases system flexibility by storing surplus renewable energy and supplying power during deficits, thus decreasing reliance on expensive grid imports and diesel generators. Renewable energy sources also reduce the cost of fuel generation and the impact on the environment. The joint utilization of BESS and renewable resources can offer cost savings, reliability improvements, and better energy balancing for interconnected microgrids.

(c) Diesel Generator

When there is a deficit in renewable energy resources, the diesel generator (DG) works as a supplementary dispatchable power source. For generators, diesel fuel is a decisive need, and the relationship between output power and cost can be defined as:

(9) $$C_{DG}(x_{DG})=c_{fuel}\;(\alpha_{DG}x_{DG}{\left(t\right)}^{2}+\beta_{DG}x_{DG}(t)+\gamma_{DG}(t))$$

where $c_{fuel}$ is the price of diesel fuel, $x_{DG}$ is described as the output power of the diesel generator, and $\left(\alpha_{DG},\beta_{DG},\gamma_{DG}\right)$ are the cost coefficients of power generation [33].

(d) Renewable Energy Resources

In order to expedite the power demand, the relationship between the output power of a wind turbine (WT) and wind speed V can be denoted as [34].

(10) $$x_{WIND}=\left\{\begin{array}{ll} 0 & V\leq v_{J}\;or\;V\geq v_{o}\\ x_{RW}\left(\frac{V-v_{J}}{v_{R}-v_{J}}\right) & v_{J}<V<v\\ x_{RW} & v_{R}\leq V<v_{o} \end{array}\right.$$

where $x_{WIND}$ and $x_{RW}$ are the actual power output and rated power of the wind generator; $v_{R}$, $v_{o}$ and $v_{J}$ are the nominal, cut-out, and cut-in wind speeds of the WT.

From [35], the power output of a PV is represented as:

(11) xPV=x′[I′+I′κ(Tpvc−Tstd)]where, I′=IactIstd

whereas $x^{\prime }$ is the power at standard temperature $T_{std}$ (i.e., 25 °C and solar irradiance $I_{std}$ (i.e., 1000 W/m^2^). $I_{act}$ is the actual solar irradiance, κ is a multiplying factor, and $T_{pvc}$ is the temperature of the PV cell.

To reduce operational costs, renewable energy resources (RERs) should be utilized fully. The total output power of PV and WT can be defined as [36]:

(12) $$x_{RE}=x_{PV}+x_{WT}$$

### 2.2. Basic Optimization Model MGs

The WT and PV are non-dispatchable sources, whereas the utility MGs, diesel generator, and battery ESS are dispatchable sources. Only dispatchable resources that are operational can be scheduled to minimize the generation cost. The minimized operational cost of the *i*th number of MG can be formulated as:

(13) $$\;\;\;\;min\;\;\;\;\;\;\;\;\;f_{i}(x_{i})=h_{i}(x_{i})+C_{MG}(x_{i}^{exc})$$

where

(14) $$\;h_{i}(x_{i})=C_{i,DG}(x_{i,DG})+C_{i,ESS}(x_{i,ESS})+C_{i,G}(x_{i,G})$$

From (14), we can rewrite (13) as:

(15) $$\min\;\;C_{i,DG}(x_{i,DG})+C_{i,ESS}(x_{i,ESS})+C_{i,G}(x_{i,G})+C_{MG}(x_{i}^{exc})$$

(16) $$\min\;\;C_{i,DG}(x_{i,DG})+C_{i,ESS}(x_{i,ESS})+C_{i,G}(x_{i,G})+C_{MG}(x_{i}^{exc})$$

(17) $$0\leq x_{i,DG}\leq x_{i,DG}^{\max}$$

(18) $$x_{i,ESS}^{chr,\max}\leq x_{i,ESS}\leq x_{i,ESS}^{dis,max}$$

(19) $$SOC_{i}^{min}\leq SOC_{i}\leq SOC_{i}^{max}$$

where $x_{i}=[x_{i,DG},x_{i,ESS},x_{i,G}]$ is the total power output of dispatchable resources (i.e., DG and ESS) and the cost of power exchanged between MG *i* and the utility grid. $C_{MG}(x_{i}^{exc})$ is the cost for exchanging power inside the distribution network. The exchange power $x_{i}^{exc}$ is positive when power is purchased from the network by MG *n*, and otherwise is negative, $x_{n,RE}$ is the total renewable energy of the MG *n*. The total load demand of the *i*th MG is denoted by $x_{i}^{d}$. Equation (16) is the power balance constraint, (17) is the DG power limit constraint, (18) is the constraint for charging and discharging limits of the battery, and (19) is the battery’s SOC maximum and minimum limit.

### 2.3. Optimization Model for Networked MGs

We assumed that under the topology of all MGs in a coalition, MGs and ECM cooperatively draw an optimal schedule and try to minimize the total operational cost of the network. Optimization of *N* interconnected MGs in a coalition through a communication network can be expressed as [37]:

(20) $$\min\;\;\;\;\;\;\;\sum_{i=1}^{N}\left(h_{i}(x_{i})+C_{i,MG}(x_{i})\right)$$

(21) $$s.t.\;\;\;\;\;\sum_{i=1}^{N}1^{T}(x_{i})=\sum_{i=1}^{N}(x_{i}^{d}-x_{i,RE})$$

where $1^{T}$ is a row. The coalition among *N* number of MGs collaboratively tries to minimize the global objective function. The optimization problem can be solved through two approaches, i.e., central and distributed. However, in this article, we will develop a distributed method to solve the problem through efficient communication.

## 3. Distributed Censored ADMM Formulation

In this section, we discuss the modeling of standard ADMM for the multi-microgrid environment. The modeled system is then updated using the concept of censored communication.

### 3.1. ADMM Protocol

ADMM is being extensively implemented in large-scale optimization problems involving multi-agent systems because of its robustness and exquisite performance in convergence. In the general form for a convex optimization problem, ADMM can be represented as [38]:

(22) min         w(x)+g(z)

(23) s.t.        Ax+Bz=c

where $x\in R^{i},\;z\;\in R^{j},\;\;A\in R^{p\times i},\;B\in R^{p\times j},c\in R^{p}.$ The x and z are optimization variables for the convex functions w and g.. Moreover, (22) is subjected to a linear constraint (23). The ADMM iteratively solves the subproblems w and g one by one to converge on optimal solutions. Furthermore, functions w and g can take any value up to +∞. The algorithm will converge to the optimal solution when w is fit with $R^{i}\to R\cup \left\{+\infty \right\}$ and g with $R^{j}\to R\cup \left\{+\infty \right\}$ [36]. The function will become zero when the constraints are not satisfied.

In order to decentralize the model designed in (20) and build coherence with the general ADMM approach, we define the following functions:

(24) $$g\left(z\right)=\left\{\begin{array}{c} +\infty z\notin R^{N}\\ 0z\in R^{N} \end{array}\right.\;\;\;\;\;\;\;\;C=\{z\in R^{N}|z_{1}+z_{2}+\cdots +z_{N}=0\}$$

Rewriting (15) to make computations simple:

(25) $$x_{i}^{exc}=1^{T}x_{i}-(x_{i}^{d}-x_{i,RE})$$

**Figure 2:**
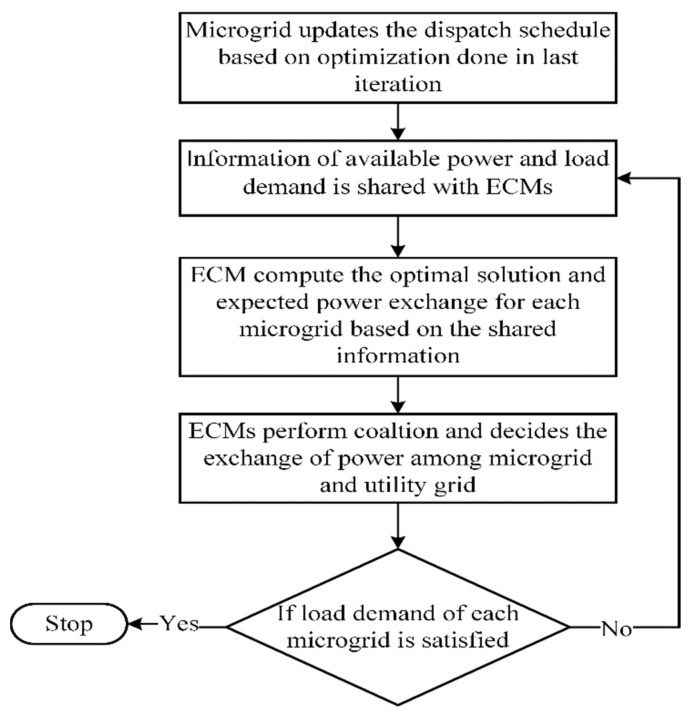
Basic algorithm for energy management of interconnected microgrids.

Following the standard form of ADMM, (20) and (13) can be re-described as:

(26) $$\min\;\;\;\;\;\;\;\sum_{i=1}^{N}h_{i}(x_{i})+g(z)$$

(27) $$s.t.\;\;\;\;\;\;\;\;\;\;\;\;{Ax}^{exc}+Bz=\;0$$

where $x^{exc}=\;[x_{1}^{exc},x_{2}^{exc},x_{3}^{exc},\ldots ,x_{N}^{exc}]$ is the vector for the expected interchangeable power of MGs in the network. The z are responsible for the consensus. The idea is to break the problem into *N* subproblems that could be solved cooperatively in a distributed manner. Following [39], the iterative ADMM process of node *i* for primal and dual variables can be defined as:

(28) $$x_{i}^{k}=\arg\;\underset{x_{i}}{min}\;h\left(x_{i}\right)+\langle x_{i}^{exc^{k-1}},\lambda_{i}^{k-1}-c\sum_{j\in N_{i}}(x_{i}^{exc^{k-1}}+x_{j}^{exc^{k-1}})\rangle \;\;+cb_{i}{\left\|x_{i}^{exc}\right\|}_{2}^{2}$$

(29) $$\lambda_{i}^{k}=\lambda_{i}^{k-1}+c\sum_{j\in N_{i}}\left(x_{i}^{exc^{k}}+x_{j}^{exc^{k}}\right)$$

where *k* is the iteration number, c>0 is the penalty parameter, and $\lambda^{k}$ is the Lagrange multiplier at the *kth* iteration. From (28), using the expected exchangeable power of MGs is required during optimization. It can also be seen in (29) that only the expected exchange is required to upgrade the Lagrange variable, which preserves the privacy of MGs. The workflow of the proposed energy management algorithm for interconnected microgrids is shown in Figure 2, including the communication and calculation processes. At the beginning, each microgrid collects local data, including load demand, renewable generation, and resource status, and transmits it to its Energy Coalition Manager (ECM). The ECM determines optimal local scheduling and forecasts power exchanges with adjacent microgrids or the utility grid. Subsequently, the ECMs work in a distributed manner and communicate iteratively via ADMM-based optimization. In each iteration, local variables are updated so that the operational costs are minimized with system constraints and power balance. The iterations repeat until convergence is achieved, which ensures the system has efficient coordination, decentralized operation, and improved system performance.

The basic optimization algorithm is shown in Figure 2. Initially, information about the available generation and load demand is shared with the ECM by each microgrid. ECMs then compute the optimal dispatch for each microgrid. The amount available for expected exchange $x_{i}^{exc}$ or, in case of a deficit, the amount to buy and expected grid exchange power $x_{G,i}$ is measured. In the next step, the ECMs of each microgrid form a coalition and try to satisfy the load demands of each when the load demand is satisfied with the algorithm.

### 3.2. Censored Communication-Based ADMM

In ADMM, each iteration comprises two phases: the first phase is the communication phase, where each node shares its local variables with adjacent neighbors, and the second phase is called the computation phase. In this phase, each node performs calculations based on the information received.

The key idea of censored communication is based on a censoring function (30) [31]. This function permits a node to share its local variables with its adjacent neighbors. The difference between ADMM and censored ADMM is that a node does not necessarily have to broadcast at each iteration; it will only transmit its local variable to its adjacent neighbors if this local variable is adequately different from the formerly transmitted one. Further, we will cover the modeling of censored ADMM for the multi-microgrid environment.

(30) $$C^{F}(k)=\left\|{\bar{x}}_{i}^{exc^{k-1}}-x_{i}^{exc^{k}}\right\|-\sigma \rho^{k}\geq 0$$

where σ>0 and ρ∈(0,1) are constants deciding the censoring threshold. In order to obtain more insight into the censoring technique, consider a state variable ${\bar{x}}_{i}^{exc^{k-1}}$. This state variable records the latest transmitted expected exchange before *k* − 1. After calculating the next state variable at *k*, the ECM of MG *i* evaluates the difference of ${\bar{x}}_{i}^{exc^{k-1}}$ and $x_{i}^{exc^{k}}$ according to (30) against the censoring threshold. ECM is permitted to transmit $x_{i}^{exc^{k}}$ if the difference is greater than the threshold and update as, ${\bar{x}}_{i}^{exc^{k}}=x_{i}^{exc^{k}}$. Otherwise, consider this message is considered “less informative” and the update proceeds as, ${\bar{x}}_{i}^{exc^{k}}={\bar{x}}_{i}^{exc^{k}-1}$. Based on this, the predesigned ADMM model is updated as:

(31) $$x_{i}^{k}=\arg\;\underset{x_{i}}{min}\;h(x_{i})+\langle x_{i}^{exc^{k-1}},\lambda_{i}^{k-1}-c\sum_{j\in N_{i}}\left({\bar{x}}_{i}^{exc^{k-1}}+{\bar{x}}_{j}^{exc^{k-1}}\right)\rangle +cb_{i}{\left\|x_{i}^{exc}\right\|}_{2}^{2}$$

(32) $$\lambda_{i}^{k}=\lambda_{i}^{k-1}+c\sum_{j\in N_{i}}\left({\bar{x}}_{i}^{exc^{k}}+{\bar{x}}_{j}^{exc^{k}}\right)$$

The algorithm for the censored ADMM follows the same procedure as described in Figure 2 with a few updates. In the coalition stage, the information exchange follows the censoring function. The optimization process will not be affected much if the Euclidean distance between previously sent and current local variable is small. However, it will reduce the communication cost (cc) by limiting the repeated exchange of information among ECMs. However, the choice of threshold can affect the optimization convergence. Moreover, a large value of σρ reduces communication cost more significantly, and setting them both to zero is the same as uncensored ADMM.

## 4. Case Study

**Figure 3:**
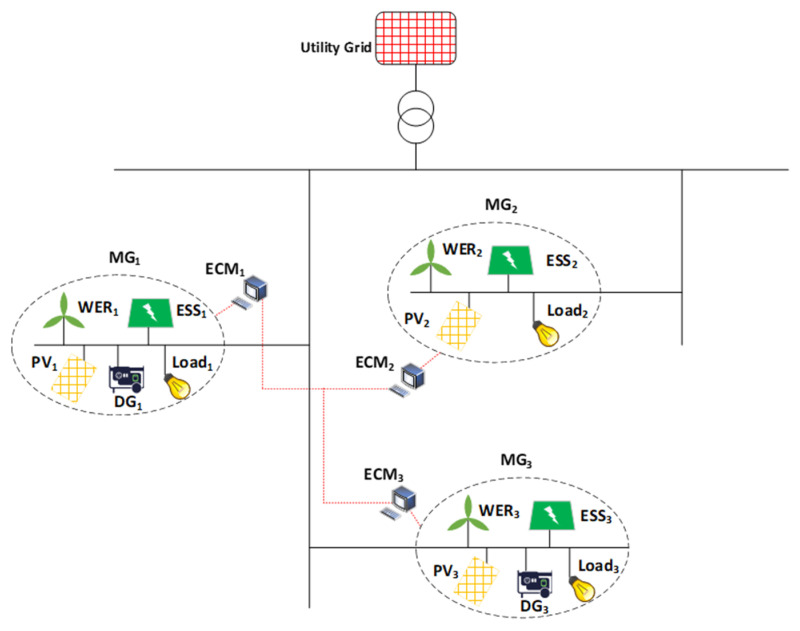
Interconnected microgrid energy sharing network for the case study.

The results demonstrate that the inclusion of BESS and renewable energy sources significantly reduces operational costs and grid dependency while improving system reliability. In the present study, the performance of the devised algorithm is assessed considering a coupled network of three microgrids (MG) integrated with the utility grid, with each MG incorporating its own Energy Control Module, as illustrated in Figure 3. The parameters of energy sources are depicted in Table 1. Each MG is composed of a load, distributed generation, an energy storage system, wind energy, and a photovoltaic (PV) system.

**Figure 4:**
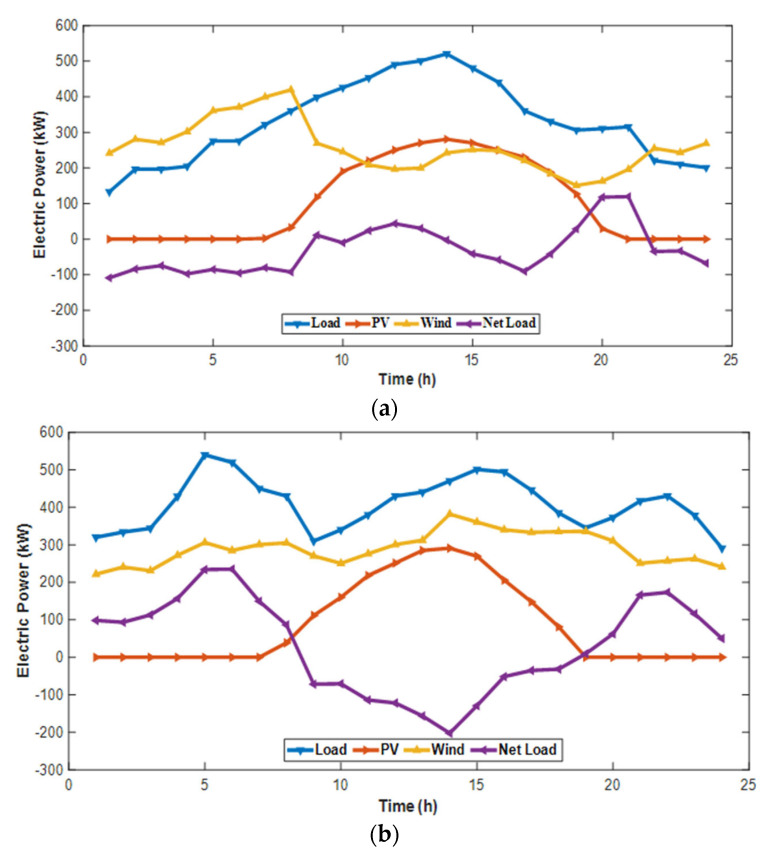

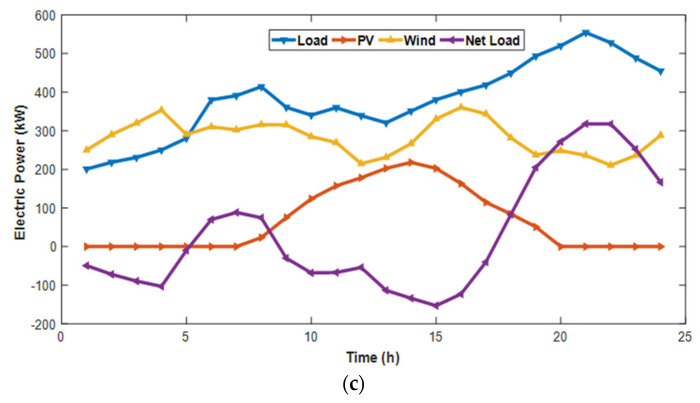
The daily profiles of load, PV, wind, and net load of three microgrids ((**a**) Microgrid 1), ((**b**) Microgrid 2), and ((**c**) Microgrid 3).

**Figure 5:**
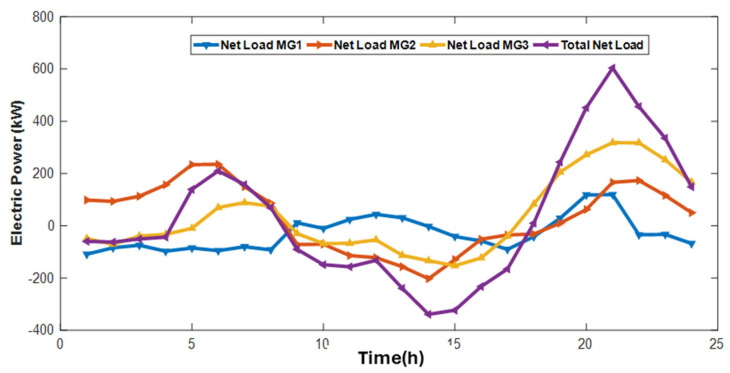
The net loads and the total net load of three microgrids.

Similarly, a positive total load means that the load is more than the total renewable power available at that time slot, and it must be supplied by the local resources, i.e., ESS and DGs, and in case of further deficit energy will be transported from other MGs/Utility grids. The power exchange between MGs and the utility is managed by the ECMs, and the DG and ESS power is consumed by the local load while promoting maximum utilization of renewable energy. In our study, the import price of electric power is kept high. The daily profile of renewable energy resources and load for the three MGs is shown in Figure 4. It can be seen from Figure 5 that renewable energy is available in excess in the middle of the day, while in the morning and evening parts of the day, it is insufficient to supply the total load demand. Initially, the scheduling is performed using standard ADMM, and then censored ADMM is used for the same scenario. The threshold parameters of censored ADMM are tuned to the best values by hit and error, i.e., σ=0.25 and ρ=0.55.

### 4.1. Optimal Scheduling Results

**Figure 6:**
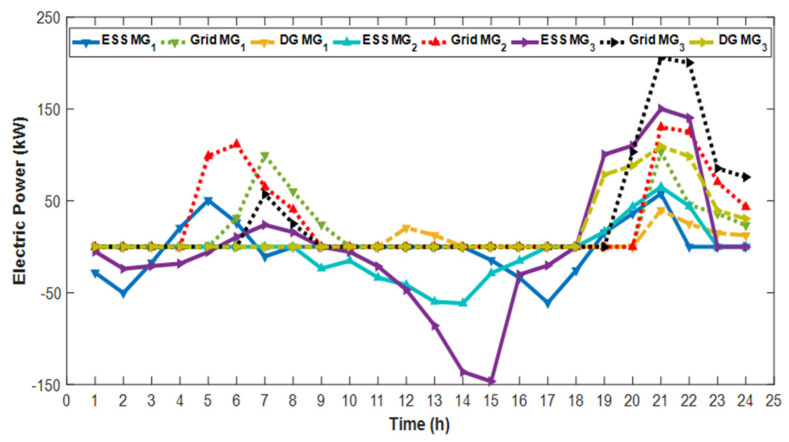
Optimal hourly schedule of energy resources and trading with the utility grid.

Figure 6 illustrates the optimal hourly schedule of energy resources. During the periods 1:00–4:00 and 8:00–17:00, there is a surplus of renewable energy, which is used for energy storage. Negative values indicate the power absorbed by the storage system.

Stored energy is used when renewable sources cannot meet demand, specifically from 5:00 to 9:00 and 19:00 to 24:00. If both renewable and storage systems cannot satisfy demand, the grid provides additional electricity. High grid power costs make distributed generation (DG) and energy storage systems (ESS) crucial for reducing grid dependency. Energy storage is prioritized in these scenarios. During the forecast period, the internal variables of three microgrids are analyzed at 21:00, with the results shown in Figure 5.

**Figure 7:**
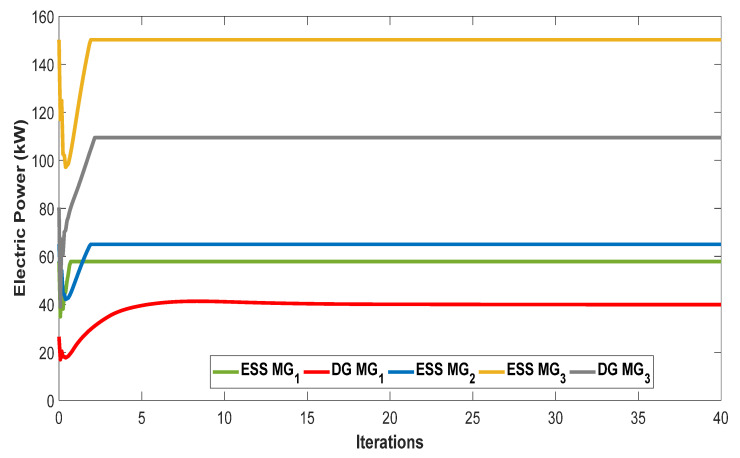
Evolution of internal variables for time slot 21.

**Figure 8:**
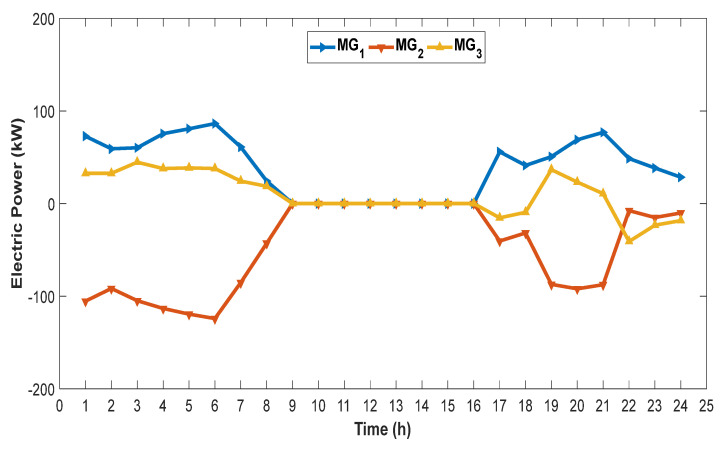
Expected power-sharing among the MGs.

The algorithm calculates power outputs for each microgrid (MG) in about 12 iterations. Figure 7 shows that total operating costs also converge within 12 iterations for 21:00. Figure 5 depicts energy storage states. During surplus periods, renewable energy is absorbed. High external energy costs ensure stored energy meets MG demand first. Excess renewable energy can be shared among MGs, as shown in Figure 8. When one MG’s renewable energy is in excess, and another requires extra power, MGs engage in power exchange. Take MG2 as an example; it shows a net positive load during the initial interval, while MG1 and MG3 both have an excess of renewable energy. As a result, MG2 takes extra power from MG1 and MG3. No power exchange occurs at noon because each MG’s renewable energy supply is sufficient. At each interval, the sum of the powers is zero, achieving power balance. The suggested distributed method efficiently and optimally meets the demand load.

### 4.2. Convergence Under Censored Admin

**Figure 9:**
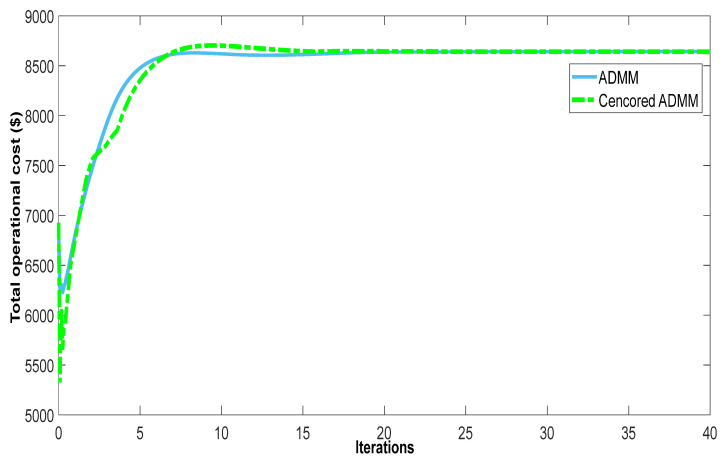
Convergence analysis of ADMM versus censored ADMM.

When comparing the two algorithms, they differ in the communication step. Nevertheless, communication costs must be considered. In simple terms, Censored ADMM demands more computation to produce optimal results. Hence, the overall operational cost will be the sole metric for comparing convergence, aiding their research. The remaining scheduling will continue in line with the algorithm’s convergence behavior. As depicted in Figure 9, we evaluated the total operational costs of both techniques for the convergence study under the same conditions for slot 21. In contrast to regular ADMM, which achieves convergence in only 12 rounds, censored ADMM takes longer, requiring 16 iterations. The primary distinction becomes evident during the communication phase, which will be elaborated further below.

### 4.3. Analysis of Computation Cost and Communication Cost

We discussed the communication costs of ADMM and censored ADMM here. To achieve this, we keep track of the total number of messages sent by each ECM. In addition, we study convergence for different levels of censoring functions. Central processing unit (CPU) time is used to measure computational cost. A machine with four 2.50 GHz Intel(R) Core (TM) i3-3120M CPUs and forty dots of random-access memory (RAM) is used to run the study in MATLAB R2019a.

For slot 21, the results are shown in Table 2. Differences may occur if the threshold value is too small. In addition, the ADMM has the highest communication costs, but it shows the fewest iterations needed to converge. The filtered ADMM, on the other hand, significantly lowers the communication cost but requires more iterations to reach convergence. Communication costs, CPU time, and iterations become worse when the threshold value of the calibrated benchmark increases. Furthermore, picking the wrong threshold might greatly increase operating cost variations. However, the communication cost of the filtered ADMM approach is still lower than that of the traditional ADMM method. Based on our analysis, filtered ADMM is a wise choice for large-scale MG networks, where expensive communication infrastructure and network capacity are major concerns.

### 4.4. Scalability Analysis

The suggested methodology is better understood after a thorough scalability examination of the two methods. Increasing the number of microgrids from three to thirty is part of this investigation.

**Figure 10:**
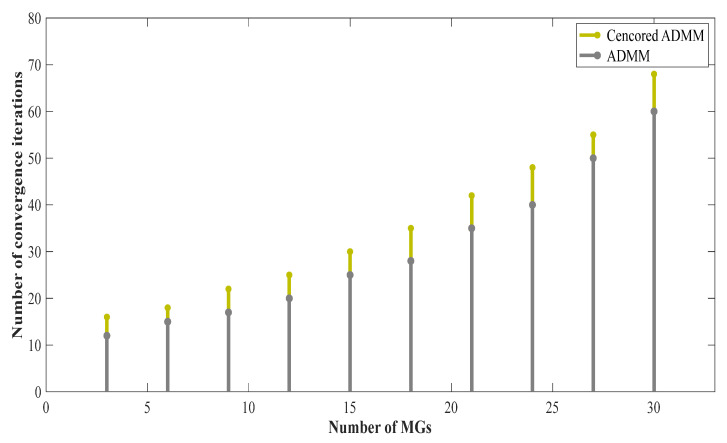
Scalability analysis and convergence iterations.

**Figure 11:**
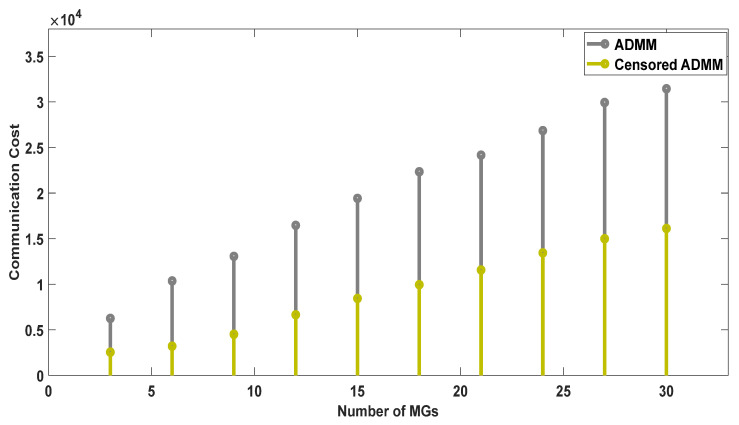
Scalability analysis and communication costs.

To increase the scale of the network, three modeled microgrids are chosen at random and then replicated. Although censored ADMM has a much greater count, the simulation results show fast iterative convergence. In Figure 10, we can see how the two methods stack up when it comes to convergence. Figure 11 shows that there is a significant benefit in terms of reducing communication costs. Since ADMM converges quickly, it follows that a higher transmission cost is required to obtain the best solution.

## 5. Conclusions

This article introduces a distributed methodology that uses ADMM and filtered ADMM techniques to solve the problem of energy management in interconnected microgrids. An energy control module (ECM) is a component of every microgrid (MG) that coordinates communication and, in a group effort, finds the best time to run the system. The suggested approach solves the issue by exchanging data only regarding power exchanges. In the event of a power outage, the system will draw from neighboring microgrids or the utility grid to make up the difference, with an emphasis on using locally stored energy and renewable energy sources that have excess. In 12 iterations for ADMM and 16 iterations for censored ADMM, the algorithm converges to the optimal solution, according to the case study. It should be noted that the censored method greatly reduces communication expenses. Similarly, the ideal solution can be easily converged upon in scalability analyses of bigger networks. The assumption of a perfect communication link, free of noise and delay, is the main constraint of the study. Subsequent studies should investigate the effectiveness of the algorithm in a real-world communication network setting.

## Figures and Tables

**Table 1:** Energy Resources Parameters.

	Energy Resources	Rated Power (kW)	Energy Storage Resource	Rated Power (kW)	Rated Capacity (kWh)
Microgrid 1	DG	200	ESS	200	600
	PV	600	-	-	-
	WER	800	-	-	-
Microgrid 2	PV	600	ESS	200	700
	WER	700	-	-	-
Microgrid 3	DG	200	ESS	300	800
	PV	500	-	-	-
	WER	800	-	-	-

**Table 2:** Analysis of computation cost and communication cost.

	σρ	Iterations	Operational Cost Deviation (10^−3^)	cc	CPU (s)
Standard ADMM	-	12	2.235	6254	1.535
Censored ADMM	0.042	not converged	-	-	-
	0.098	18	3.854	2725	2.725
	0.137	16	2.424	2546	2.465
	0.564	22	4.568	3915	3.564
	0.896	32	6.241	5584	5.954

## Data Availability

The original contributions presented in this study are included in the article. Further inquiries can be directed to the corresponding author.
